# Accuracy of low-density lipoprotein cholesterol estimation at very low levels

**DOI:** 10.1186/s12916-017-0852-2

**Published:** 2017-04-20

**Authors:** Renato Quispe, Aditya Hendrani, Mohamed B. Elshazly, Erin D. Michos, John W. McEvoy, Michael J. Blaha, Maciej Banach, Krishnaji R. Kulkarni, Peter P. Toth, Josef Coresh, Roger S. Blumenthal, Steven R. Jones, Seth S. Martin

**Affiliations:** 1 Ciccarone Center for the Prevention of Heart Disease, Division of Cardiology, Department of Medicine, Johns Hopkins University School of Medicine, 600 N. Wolfe Street, Carnegie 591, Baltimore, MD 21287 USA; 2 Department of Medicine, Medstar Good Samaritan/Union Memorial Hospital, Baltimore, MD USA; 3 Department of Cardiovascular Medicine, Cleveland Clinic, Cleveland, OH USA; 4 Welch Center for Prevention, Epidemiology, and Clinical Research, Department of Epidemiology, Johns Hopkins Bloomberg School of Public Health, Baltimore, MD USA; 5 Department of Hypertension, Chair of Nephrology and Hypertension, Medical University of Lodz, Lodz, Poland; 6 Atherotech Diagnostics Laboratory, Birmingham, AL USA; 7 Department of Preventive Cardiology, CGH Medical Center, Sterling, IL USA; 8 University of Illinois College of Medicine, Peoria, IL USA

**Keywords:** Low-density lipoprotein cholesterol, Very low, Accuracy, Friedewald estimation, Novel method, Clinical decision making

## Abstract

**Background:**

As the approach to low-density lipoprotein cholesterol (LDL-C) lowering becomes increasingly intensive, accurate assessment of LDL-C at very low levels warrants closer attention in individualized clinical efficacy and safety evaluation. We aimed to assess the accuracy of LDL-C estimation at very low levels by the Friedewald equation, the de facto clinical standard, and compare its accuracy with a novel, big data-derived LDL-C estimate.

**Methods:**

In 191,333 individuals with Friedewald LDL-C < 70 mg/dL, we compared the accuracy of Friedewald and novel LDL-C values in relation to direct measurements by Vertical Auto Profile ultracentrifugation. We examined differences (estimate minus ultracentrifugation) and classification according to levels initiating additional safety precautions per clinical practice guidelines.

**Results:**

Friedewald values were less than ultracentrifugation measurement, with a median difference (25th to 75th percentile) of –2.4 (–7.4 to 0.6) at 50–69 mg/dL, –7.0 (–16.2 to –1.2) at 25–39 mg/dL, and –29.0 (–37.4 to –19.6) at < 15 mg/dL. The respective values by novel estimation were –0.1 (–1.5 to 1.3), –1.1 (–2.5 to 0.3), and –2.7 (–4.9 to 0.0) mg/dL. Among those with Friedewald LDL–C < 15, 15 to < 25, and 25 to < 40 mg/dL, the classification was discordantly low in 94.9%, 82.6%, and 59.9% of individuals as compared with 48.3%, 42.4%, and 22.4% by novel estimation.

**Conclusions:**

Estimation of even lower LDL-C values (by Friedewald and novel methods) is even more inaccurate. More often than not, a Friedewald value < 40 mg/dL is underestimated, which translates into unnecessary safety alarms that could be reduced in half by estimation using our novel method.

**Electronic supplementary material:**

The online version of this article (doi:10.1186/s12916-017-0852-2) contains supplementary material, which is available to authorized users.

## Background

The critical importance of low-density lipoprotein cholesterol (LDL-C) lowering in cardiovascular disease management and prevention of atherothrombotic events is well established [1–7]. LDL-C is the lipid parameter that is most widely used to guide clinical decision making. Most major international guidelines endorse an LDL-C target of < 70 mg/dL for high-risk patients [8–10] and the IMProved Reduction of Outcomes: Vytorin Efficacy International Trial (IMPROVE-IT) trial [11] showed additional benefit from attaining even lower LDL-C levels (~50 mg/dL) by adding ezetimibe to statin therapy. As more aggressive LDL-C lowering approaches become increasingly common, with use of human monoclonal antibodies targeting proprotein convertase subtilisin-kexin type 9 (PCSK9) [12–14], the accuracy of LDL-C assessment at very low levels gains importance.

There is little experience in managing patients with very low LDL-C and considerable concern has arisen about whether the benefit-to-risk ratio for lowering LDL-C holds at these very low levels. Thus far, safety data have been reassuring with respect to achievement of very low LDL-C with PCSK9 inhibitors. However, there remains a need for longer term safety data in humans with low LDL-C levels outside of those with genetically low LDL-C [15, 16]. With a median follow-up of 6 years, IMPROVE-IT provides the most important and reassuring data to date [11]. The 2013 ACC/AHA cholesterol guidelines recommended considering statin dose down-titration at LDL-C < 40 mg/dL [17]. In phase 3 trials of some PCSK9 inhibitors, active safety monitoring was closely performed if on-treatment LDL-C levels were < 25 mg/dL, and medication was stopped at < 15 mg/dL [18]. In the ODYSSEY OUTCOMES trial, the investigators are specifically avoiding LDL-C levels < 15, adjusting alirocumab to achieve LDL-C 15 to < 50 mg/dL. The Food and Drug Administration (FDA) recommended close monitoring for adverse events at these very low LDL-C levels in order to track any potential long-term safety effects [14, 18]. Therefore, accurate assessment of LDL-C at very low levels is directly tied to the accuracy of therapeutic dosing and the attention to safety monitoring.

No accurate direct LDL-C assay is widely available in clinical practice. Direct chemical assays of LDL-C, though widely available, have repeatedly shown inaccurate results [19–22]. Ultracentrifugation is the gold standard for direct measurement, but is resource intensive and available in few laboratories. Thus, Friedewald-estimation of LDL-C (LDLf-C), derived from 448 patients in 1972 [23], is the de facto clinical standard. The Friedewald method converts triglycerides (TG) to very-low density lipoprotein cholesterol (VLDL-C) assuming a fixed factor of 5 for the TG:VLDL-C ratio (in mg/dL).

Friedewald estimation generally provides an accurate result in patients with moderate and high LDL-C levels and well-controlled TG levels (<150 mg/dL). However, in patients with low LDL-C, a group largely outside the range of the Friedewald derivation sample, and elevated TG, the Friedewald equation tends to significantly underestimate LDL-C levels [24, 25]. A small study recently suggested that this problem may be magnified in individuals with very low LDL-C levels [26].

In contrast to the Friedewald method, our group previously used an unbiased big data approach to develop a novel method, published in 2013 [27], that accounts for patient heterogeneity by incorporating an adjustable TG:VLDL-C ratio [27]. The adjustable factors were derived from our previously reported 180-cell method, whereby TG and non-HDL-C were used to determine patient-specific flexible ratios to estimate VLDL-C. This novel method appears to provide a more accurate estimate, but its comparative performance has not been specifically assessed throughout a range of clinically relevant very low LDL-C levels, and in particular, at LDL-C less than 70 mg/dL.

Therefore, in a large contemporary population with very low LDL-C levels (< 70 mg/dL), we aimed to assess and compare the accuracy of Friedewald and novel LDL-C estimation in absolute terms and according to clinical categories.

## Methods

### Study population

We examined consecutive lipid profiles from US patients aged 18 years or older in the Very Large Database of Lipids, which has lipid distributions that are similar to the National Health and Nutrition Examination Survey population (NHANES) 2007–2008 [28]. Each patient had a Vertical Auto Profile (VAP, Atherotech Diagnostics Lab, Birmingham, Alabama) from 2009 to 2011 for clinical reasons. We excluded patients with TG ≥ 400 mg/dL, according to specifications of the Friedewald equation [23].

The Johns Hopkins institutional review board waved the requirement of informed consent and declared our study exempt as we used only de-identified data routinely collected during clinical lipid determinations. The Very Large Database of Lipids and its studies are registered on clinicaltrials.gov (NCT01698489). This is phase C of study 1 (VLDL-1C).

### Lipid measurements

The VAP test separates lipoproteins by density gradient ultracentrifugation, then uses a colorimetric method of cholesterol determination [29]. This methodology has been developed using fractions from sequential flotation method [30, 31]. The accuracy and precision of VAP lipid parameters have been validated against β-quantification at Washington University’s Core Laboratory for Clinical Studies (St. Louis, Missouri), showing a correlation coefficient (R) of 0.980 for LDL-C [29]. Of note, the VAP method was subjected to a new lab validation, in which an experiment was performed by running VAP assays using serially increasing amounts of pooled serum (5–200 μL). LDL-C was very linear throughout the tested range of 9 to 520 mg/dL. On the other hand, TG levels were directly measured using the Abbott ARCHITECT C-8000 system (Abbott Laboratories, Abbott Park, Illinois). Further details about the validation of VAP lipid parameters have been previously described [29].

### Study variables

Directly-measured LDL-C (LDLd-C) via ultracentrifugation was considered the gold standard. LDLf-C was estimated as total cholesterol minus HDL-C minus TG/5, in mg/dL. Novel LDL-C (LDLn-C) was estimated using 1 of 180 different factors for the TG/VLDL-C ratio, according to non-HDL-C and TG levels [27]. This method has undergone independent external validation by groups inside and outside the US, and showed improvements over Friedewald estimation in those studies [32, 33].

### Statistical analysis

First, we used scatter plots and Bland–Altman plots to visually assess discordance between LDL-C values and LDLd-C in the overall population and by TG categories. We also calculated correlation between LDL-C values and direct LDL-C through spearman’s Rho. Next, we calculated differences between LDL-C values and LDLd-C (LDLf-C minus LDLd-C and LDLn-C minus LDLd-C), so that negative values represent underestimation, and positive values represent overestimation. Medians (25–75th percentile) were calculated and compared using the Kruskal–Wallis test. Since the performance of Friedewald estimation is TG dependent, we also performed analyses by three TG categories: < 150, 150–199, and 200–399 mg/dL.

Additionally, according to clinically-relevant LDL-C categories, we determined proportions of concordance between estimated and directly-measured LDL-C. When discordance was present, we labeled an LDL-C estimate as discordantly low if it was in a lower category than LDLd-C, and vice versa for discordantly high. Cohen’s Kappa statistics (95% CI) were calculated for agreement between values and direct LDL-C measurement. The five LDL-C cut-off points used to define categories were: 15, 25, 40, 50, and 70 mg/dL, corresponding to <0.1th, 0.2th, 1.3th, 3.6th, and 14.7th percentiles in our study population, respectively. The 15 and 25 mg/dL levels were selected based on their use in clinical trial protocols of PCSK9 inhibitors, the 40 mg/dL cut-off point because of the class IIb recommendation in the ACC/AHA guidelines to consider statin down-titration below this level, the 50 mg/dL level as this is an emerging clinical target based on the IMPROVE-IT trial, and 70 mg/dL as an established goal in multiple guidelines.

Statistical analyses of numerical data were performed in Stata version 13.0 (StataCorp LP, College Station, Texas). Logarithmically scaled pseudocolor-encoded data density plots were generated in R Version 2.15.1 (Vienna, Austria) using the IDPmisc package.

## Results

### Patient characteristics

In 1,310,440 patients with TG < 400 mg/dL, 191,333 had LDLf-C < 70 mg/dL, 153,917 had LDLn-C < 70 mg/dL, and 154,725 had LDLd-C < 70 mg/dL (Table 1). Among patients with LDLf-C < 70 mg/dL, the proportion with TG levels 200–399 mg/dL increased as LDLf-C levels decreased, reaching 81.3% in patients with LDLf-C < 15 mg/dL. In contrast, only 16.9% of those with LDLn-C < 15 mg/dL and 4.4% with LDLd-C < 15 mg/dL had TG levels of 200–399 mg/dL. In patients with LDLd-C < 70 mg/dL, the vast majority had TG levels < 150 mg/dL (Table 1).

**Table 1:** Distribution of individuals with very low LDL-C levels across LDL-C and TG categories

TG (mg/dL)	Friedewald-estimated LDL-C (mg/dL)					
	< 15	15 to < 25	25 to < 40	40 to < 50	50 to < 70	Total
< 150	82 (9.9)	716 (33.0)	7591 (52.9)	18,986 (64.6)	103,096 (71.3)	130,471 (68.2)
150–199	73 (8.8)	325 (15.0)	2370 (16.5)	4504 (15.3)	20,485 (14.2)	27,757 (14.5)
200–399	672 (81.3)	1126 (52.0)	4385 (30.6)	5918 (20.1)	21,004 (14.5)	33,105 (17.3)
Total	827 (0.4)	2167 (1.1)	14,346 (7.5)	29,408 (15.4)	144,585 (75.6)	191,333
TG (mg/dL)	Novel method-estimated LDL-C (mg/dL)					
	< 15	15 to < 25	25 to < 40	40 to < 50	50 to < 70	Total
< 150	90 (76.3)	638 (81.3)	7067 (83.3)	17,708 (82.9)	98,868 (80.3)	124,371 (80.8)
150–199	8 (6.8)	71 (9.0)	788 (9.3)	2146 (10.1)	13,157 (10.7)	16,170 (10.5)
200–399	20 (16.9)	76 (9.7)	630 (7.4)	1506 (7.0)	11,144 (9.0)	11,144 (98.7)
Total	118 (0.1)	785 (0.5)	8485 (5.5)	21,360 (13.9)	123,169 (80.0)	153,917
TG (mg/dL)	Directly-measured LDL-C (mg/dL)					
	< 15	15 to < 25	25 to < 40	40 to < 50	50 to < 70	Total
< 150	64 (94.1)	500 (85.8)	6328 (82.4)	16,744 (81.0)	100,149 (79.7)	123,785 (80.0)
150–199	1 (1.5)	42 (7.2)	704 (9.2)	2121 (10.3)	14,347 (11.4)	17,215 (11.1)
200–399	3 (4.4)	41 (7.0)	648 (8.4)	1803 (8.7)	11,230 (8.9)	13,725 (8.9)
Total	68 (< 0.1)	583 (0.4)	7680 (5.0)	20,668 (13.3)	125,726 (81.3)	154,725

Numbers shown are n with column percentages in parentheses

### Correlation between LDL-C values and direct ultracentrifugation measurement

**Fig. 1:**
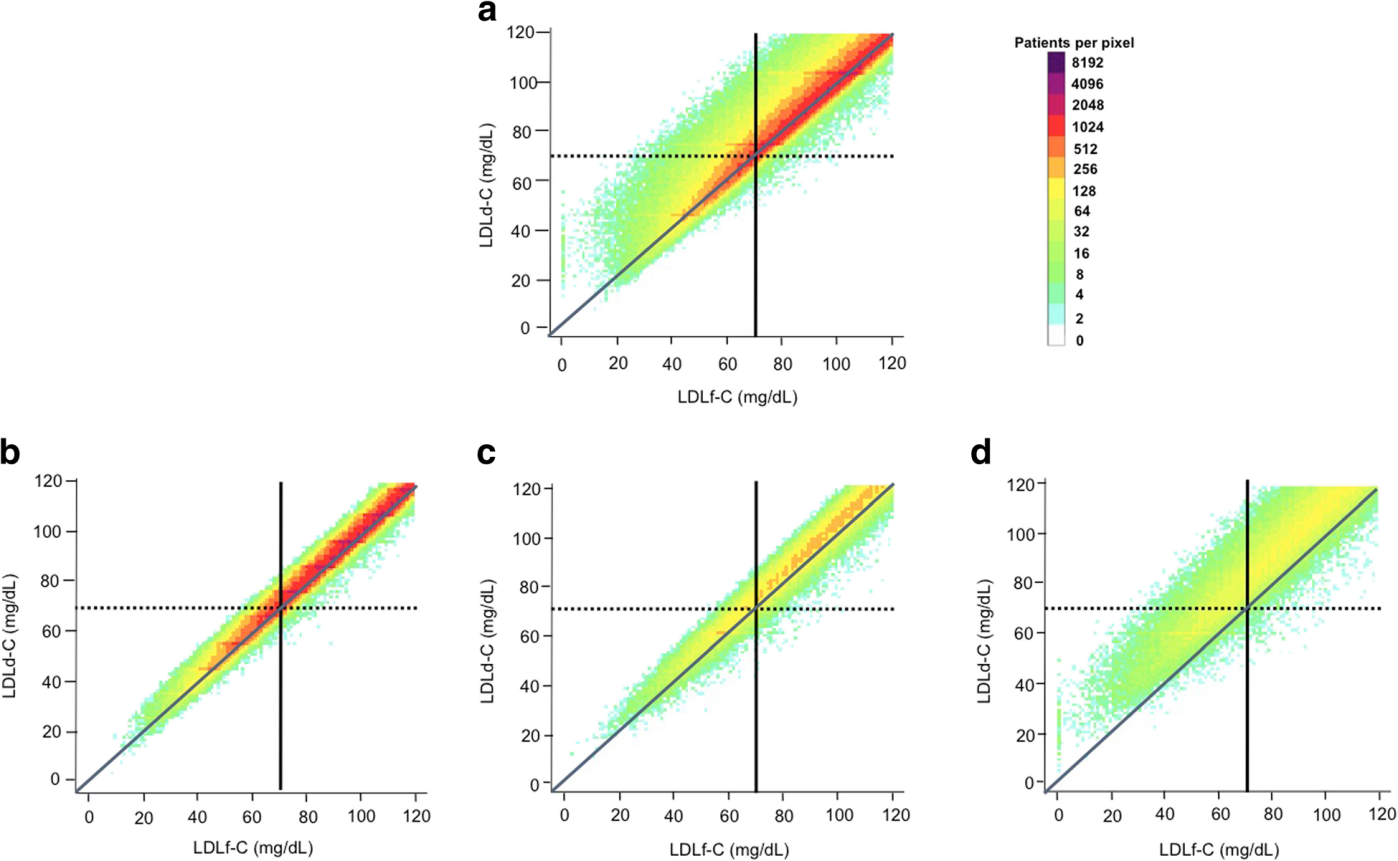
Discordance between direct ultracentrifugation measured and Friedewald-estimated LDL-C. Levels of LDLd-C (vertical axis) and LDLf-C (horizontal axis) are presented on this logarithmically scaled color density plot, with increasing density from light blue to purple. *Panel* ***a*** *:* Overall population; *Panel* ***b*** *:* TG < 150 mg/dL; *Panel* ***c*** *:* TG 150–199 mg/dL; *Panel* ***d*** *:* TG 200–399 mg/dL. A line of unity is included as well as lines indicating LDL-C 70 mg/dL. LDLd-C: directly-measured LDL-C by ultracentrifugation; LDLf-C: Friedewald-estimated LDL-C

Overall, LDLd-C appeared more strongly correlated with LDLn-C (Rho = 0.9401, *P* < 10^–15^) than with LDLf-C (Rho = 0.7408, *P* < 10^–15^) at LDL-C < 70 mg/dL (Figs. 1a vs. 2a). This difference in correlation was more apparent at higher TG levels (Figs. 1b–d vs. 2b–d). Bland-Altman plots also showed a higher agreement of LDLd-C with LDLn-C, than with LDLf-C (Additional file 1: Figure S1).

**Fig. 2:**
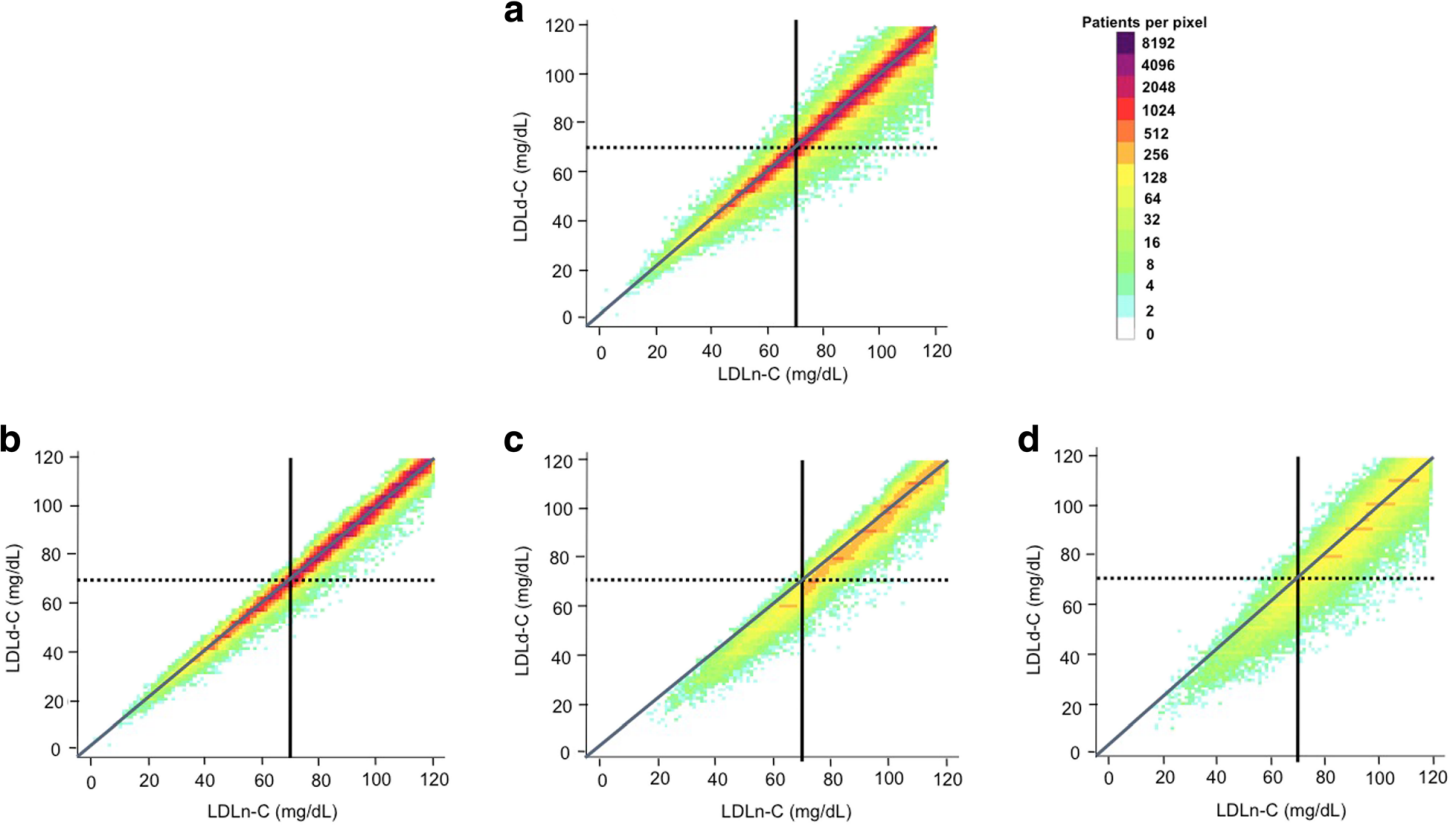
Discordance between direct ultracentrifugation measured and novel method-estimated LDL-C. Levels of LDLd-C (vertical axis) and LDLn-C (horizontal axis) are presented on this logarithmically scaled color density plot, with increasing density from light blue to purple. *Panel* ***a*** *:* Overall population; *Panel* ***b*** *:* TG < 150 mg/dL; *Panel* ***c*** *:* TG 150–199 mg/dL; *Panel* ***d*** *:* TG 200–399 mg/dL. A line of unity is included as well as lines indicating LDL-C 70 mg/dL. LDLd-C: directly-measured LDL-C by ultracentrifugation; LDLn-C: Novel method-estimated LDL-C

### Differences between LDL-C values and direct ultracentrifugation measurement

Friedewald values were lower than direct measurement, with a median difference (25–75th) of –2.4 (–7.4 to 0.6) at 50–69 mg/dL, –7.0 (–16.2 to –1.2) at 25–39 mg/dL, and –29.0 (–37.4 to –19.6) at < 15 mg/dL. Respective values by novel estimation were –0.1 (–1.5 to 1.3), –1.1 (–2.5 to 0.3), and –2.7 (–4.9 to 0.0) mg/dL (Table 2). Differences were larger at higher TG levels. For example, at LDLf-C 50–69 mg/dL, the difference was –0.6 (–3.2 to 1.4) mg/dL at TG < 150, and –17.0 (–22.6 to –12.4) mg/dL at TG 200–399. This TG-dependent effect was not seen using novel estimation with respective values of –0.2 (–1.4 to 1.1) and –0.2 (–3.8 to 3.6). Medians were significantly different across groups (*P* < 0.0001).

**Table 2:** Difference of LDL-C estimates relative to direct ultracentrifugation by LDL-C and TG categories

	Overall population		TG < 150 mg/dL		TG 150–199 mg/dL		TG 200–399 mg/dL	
Estimated LDL-C (mg/dL)	LDLf-C	LDLn-C	LDLf-C	LDLn-C	LDLf-C	LDLn-C	LDLf-C	LDLn-C
< 15	–29.0 –37.4 to –19.6	–2.7 –4.9 to 0.0	–3.8 –6.8 to 0.0	–1.5 –3.2 to 0.4	–15.0 –17.2 to –11.4	–8.2 –9.6 to –5.8	–32.5 –39.0 to –25.6	–10.8 –15.3 to –6.9
15 to < 25	–16.2 –26.8 to –5.8	–1.8 –3.5 to –0.3	–2.8 –6.0 to 0.4	–1.5 –2.7 to 0.0	–13.0 –15.4 to –10.6	–4.6 –7.0 to –1.3	–26.1 –33.2 to –20.4	–6.7 –10.3 to –3.3
25 to < 40	–7.0 –16.2 to –1.2	–1.1 –2.5 to 0.3	–1.6 –4.8 to 0.8	–1.0 –2.2 to 0.3	–11.0 –13.6 to –8.2	–2.7 –4.9 to –0.3	–21.8 –28.8 to –16.6	–3.2 –7.0 to 0.4
40 to < 50	–4.0 –10.6 to –0.2	–0.7 –2.0 to 0.7	–1.2 –4.0 to 1.2	–0.7 –1.9 to 0.6	–9.8 –12.4 to –7.2	–1.1 –3.3 to 1.3	–19.4 –25.4 to –14.6	–1.4 –4.7 to 2.1
50 to < 70	–2.4 –7.4 to 0.6	–0.1 –1.5 to 1.3	–0.6 –3.2 to 1.4	–0.2 –1.4 to 1.1	–8.6 –11 to –6.0	0.0 –2.1 to 2.6	–17.0 –22.6 to –12.4	–0.2 –3.8 to 3.6
Total	–2.8 –8.6 to 0.4	–0.3 –1.7 to 1.1	–0.8 –3.4 to 1.4	–0.3 –1.5 to 1.0	–9.0 –11.6 to –6.2	–0.3 –2.4 to 2.3	–18.4 –24.6 to –13.6)	–0.5 –4.1 to 3.3

Differences reported as median above with 25th to 75th percentiles below, in mg/dL. Differences were calculated as: LDLf-C – LDLd-C, and LDLn-C – LDLd-C; thus, negative values indicate underestimation and vice versa. Numbers of individuals per LDL-C category are the same as in Table 1

### Discordance of individuals across clinically relevant, very low LDL-C categories

Almost one in every four individuals (22.9%) with LDLf-C < 70 mg/dL, but only one in 16 (6.3%) with LDLn-C < 70 mg/dL, had LDLd-C ≥ 70 mg/dL. Discordantly high LDL-C values were uncommon (0.9% for LDLn-C and 0.6% for LDLf-C).

The proportion of individuals with discordantly low LDLf-C increased at lower levels and, more often than not, those with LDLf-C < 40 mg/dL had LDLd-C in a higher clinical category (Table 3). For the < 15, 15 to < 25, 25 to < 40, 40 to < 50, and 50 to < 70 mg/dL LDLf-C categories, discordantly low proportions were 94.9%, 82.6%, 59.9%, 52.7%, and 28.7%, respectively. Cohen’s Kappa index was 0.746 (0.746–0.747, *P* < 0.00001).

**Table 3:**
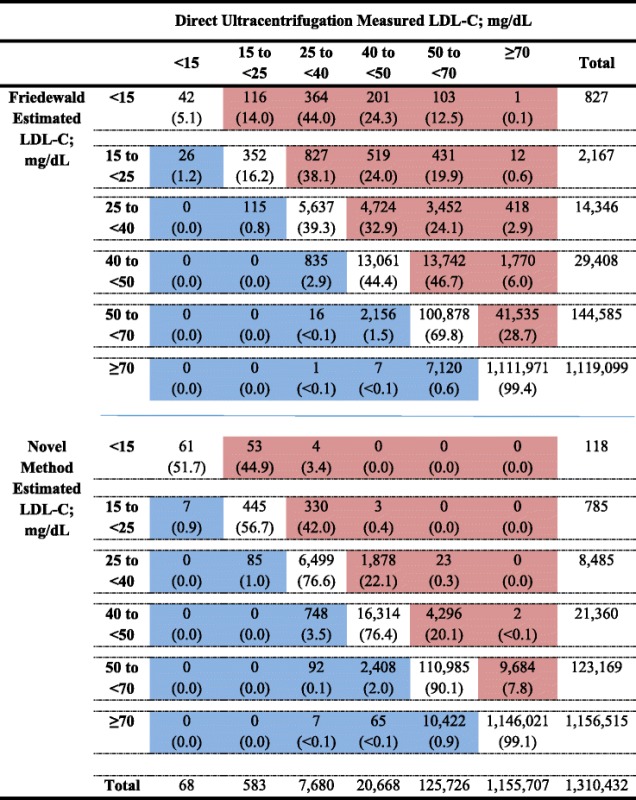
Proportions of concordance between estimation methods and direct ultracentrifugation LDL-C

Numbers shown are n above with row percentages in parentheses below

White cells: concordance; Blue cells: discordantly high; Red cells: discordantly low

On the other hand, concordance between LDLn-C and LDLd-C was significantly higher. The discordant proportion was never greater than 50% at these very low LDL-C categories. For the < 15, 15 to < 25, 25 to < 40, 40 to < 50, and 50 to < 70 mg/dL categories, discordantly low proportions were 48.3%, 42.4%, 22.4%, 20.1%, and 7.8%, respectively. Cohen’s Kappa index was 0.892 (0.891–0.893, *P* < 0.00001).

When LDLn-C was discordantly low, it was nearly always low by a difference of one clinical category (i.e., 40 to < 50 and 50 to < 70 mg/dL). In contrast, when LDLf-C was low, it was frequently low by 2 or even 3 clinical categories as compared to ultracentrifugation measured LDL-C.

### Discordance across LDL-C categories by TG levels

Having a discordantly low LDL-C estimate was significantly more common at higher TG levels, reaching up to 39.0% and 59.4% for LDLf-C, and 7.8% and 16.6% for LDLn-C at TG 150–199 and 200–399 mg/dL, respectively. Of note, in patients with TG levels of 150–199 and 200–399 mg/DL, the number of individuals with LDLf-C < 15 mg/dL but LDLd-C ≥ 15 mg/dL (Additional file 2: Table S1) was approximately 9 and 30 times higher, respectively, compared to those with LDLn-C < 15 mg/dL but LDLd-C ≥ 15 mg/dL (Additional file 3: Table S2).

## Discussion

In an analysis comprising a uniquely large number of individuals with very low LDL-C levels, our data show that estimation of even lower LDL-C levels is even more inaccurate. This study expands upon our prior work in the Very Large Database of Lipids [24] by closely examining the accuracy of LDL-C within the very low range. More often than not, LDL-C levels estimated by the Friedewald equation are classified falsely low if < 40 mg/dL, when accurate safety monitoring is most needed, and this proportion exceeds 80% at TG ≥ 150 mg/dL. Although estimation of LDL-C remains imperfect by the novel method, it provides a substantially more accurate estimation than the Friedewald equation at very low LDL-C levels, halving the proportion of falsely low classifications.

### Implications for efficacy and safety assessment

The IMPROVE-IT trial [11] testing the addition of ezetimibe to statin therapy helped confirm the “the lower the better” hypothesis and supports aiming for lower LDL-C levels if it can preserve a favorable risk-benefit ratio [34]. Moreover, recently FDA approved monoclonal antibodies to PCSK9 appear safe through 1 year and robustly lower LDL-C [35]. The addition of alirocumab and evolocumab to standard of care in the ODYSSEY LONG-TERM and Open Label Study of Long Term Evaluation Against LDL-C Trial (OSLER) studies, respectively, yielded mean LDL-C reductions of approximately 60% to levels of approximately 50 mg/dL and preliminary short-term outcome data show an incremental 50% relative reduction in cardiovascular events compared to standard of care [12, 13]. A meta-analysis found that PCSK9 inhibitors reduced all-cause mortality (OR, 0.45; CI, 0.23–0.86) [35], and long-term outcome trials are eagerly awaited [36–39].

An eligibility criterion for those long-term trials is an on-treatment LDL-C ≥ 70 mg/dL. Based on our data, 29% of persons with Friedewald LDL-C levels of 50–69 mg/dL actually have a directly-measured LDL-C ≥ 70 mg/dL. Therefore, individuals may be excluded from the long-term trials because of an underestimated LDL-C level. These trials are focused on high-risk patients, one feature of which is a concurrently high TG level, a setting wherein LDL-C underestimation is more likely to occur by Friedewald estimation.

For those patients who do qualify for trial participation or receive therapies in routine practice to treat their LDL-C down to very low levels, extra concern over ensuring appropriate risk-benefit ratio is warranted. This issue was raised in recent FDA advisory deliberations on PCSK9 inhibitors and some PCSK9 inhibitor trial protocols included active safety monitoring for LDL-C levels < 25 mg/dL and drug discontinuation when LDL-C was < 15 mg/dL [14, 18]. Since these LDL-C cut-off points were not derived from Friedewald LDL-C values, our findings raise the question of potential misinformed decision making due to LDL-C underestimation when relying on the Friedewald equation. This might translate into undue anxiety, increased resource utilization (e.g., clinic visits, additional lab work), and inappropriate therapeutic adjustment.

The association between very low LDL-C levels and adverse events has been controversial. Although the Pravastatin or Atorvastatin Evaluation and Infection Therapy (PROVE-IT) study demonstrated augmented risk reduction without safety concerns among participants with LDL-C < 40 mg/dL [40], a post-hoc analysis of the Justification for the Use of Statins in Primary Prevention: An Intervention Trial Evaluating Rosuvastatin (JUPITER) trial showed a significantly higher incidence of new-onset diabetes, hematuria, and hepatobiliary disorders in rosuvastatin-treated participants with LDL-C ≤ 30 mg/dL as compared to rosuvastatin-treated participants with LDL-C > 30 mg/dL and placebo-allocated participants [41]. Of note, LDL-C was estimated by Friedewald equation, and therefore more than 50% of values were discordantly low based on our results. In a recent meta-analysis, PCSK9 therapy was associated with a significant increase in neurocognitive adverse events compared with placebo, although this number was yet small [16]. However, this preliminary finding is still undergoing further investigation.

Several PCSK9 trials directly measured LDL-C by ultracentrifugation at different time points to support treatment effect data obtained using the Friedewald equation [14]. In trials of alirocumab [13] and evolocumab [42], the placebo-subtracted percentage change in LDLf-C was 2–4% greater than that of direct LDL-C, consistent with modest group averaged LDL-C underestimation at lower LDL-C levels in follow-up (in patients with generally well controlled TG levels). However, actual difference between LDLf-C and direct LDL-C in the patient was not evaluated as we have done in this study. While accurate measurement of LDL-C is important in accurately assessing group averaged treatment effects in clinical trials, it is also important for accurate patient-level monitoring of adverse events at very low LDL-C levels (< 25 mg/dL) as suggested by the FDA [14]. For example, approximately 40% and 26% of trial participants using alirocumab [18] and evolocumab [42], respectively, had Friedewald-estimated LDL-C levels < 25 mg/dL. Applying the findings from this study, we can estimate that approximately four in five patients actually could have had LDL-C levels ≥ 25 mg/dL.

As clinical practice moves towards lower LDL-C levels than ever before with the availability of new cholesterol lowering drugs, our findings will tend to have greater clinical relevance. The size of the present study and methodological approach, detailing accuracy of estimation across multiple clinically relevant reference categories, adds to prior work. We suggest that an update to the current de facto LDL-C assessment will likely be crucial for personalized clinical decision-making, clinical trial design, and adverse event monitoring and prevention.

### Potential alternatives to Friedewald LDL-C

If an accurate method for directly measuring LDL-C was widely available, or at least widely scalable, that could be a simple solution, assuming reasonable cost. However, no such method exists. Since the introduction of the Friedewald equation, multiple chemical based assays for direct LDL-C measurement have been introduced, but do not appear to provide an improvement [19–22]. These assays show non-specificity toward abnormal lipoproteins and fail to meet accuracy standards in diseased individuals. While ultracentrifugation was used to assess some participant samples in PCSK9 inhibitor trials, this was for research purposes only and cannot be practically implemented in clinical practice, as noted in the FDA proceedings [40].

In this context, a more accurate estimate of LDL-C is desirable from both a cost and accuracy perspective. Although multiple other groups have proposed alternative methods for LDL-C estimation, our novel LDL-C estimation appears most accurate [27] and is best validated. The performance is consistent across TG levels, the main component of the lipid profile that varies with fasting, and novel LDL-C requires no additional testing. The method can be implemented by incorporation into laboratory information technology systems for automated reporting, via Excel and Stata software available for free download at ldlcalculator.com, or via the Johns Hopkins LDL-C Calculator smartphone app that is freely available for iOS and Android.

While LDL-C is the focus of most clinical practice guidelines and clinical trials, non-HDL-C and apolipoprotein B are also included in some guidelines and warrant consideration for guiding treatment. However, clinicians are not as familiar with these [39] and clinically relevant reference values for efficacy and safety assessment at very low levels have not been established. Moreover, their responsiveness to more intensive lipid-lowering agents like PCSK9 inhibitors differs from LDL-C.

### Limitations

Limitations of our database have been discussed previously in detail [27], the main limitation being the lack of clinical, medication, or demographic information other than age and sex. However, lipid distributions closely match a nationally representative US survey (NHANES) [27]. Individuals with TG < 400 mg/dL were excluded in this study as it is well-known that LDL-C estimation is highly inaccurate in this setting and the clinical priority is managing hypertriglyceridemia. Moreover, the samples in this study were obtained for clinical purposes and thereby include a mix of fasting and non-fasting samples. Friedewald estimation may have performed better if only fasting samples had been included; however, inclusion of non-fasting assessments is representative of current clinical practice in Europe [43] and the US [44]. Given that novel LDL-C estimation showed more stable performance across TG levels, it may be more suitable for both fasting and non-fasting lipid assessment. Finally, external validation of our results are required; in particular, similar analyses would be of interest in patients treated with high-intensity statin therapy with or without PCSK9 inhibitors given the high proportion of individuals with very low LDL-C levels and the availability of direct ultracentrifugation measurement in subsamples of trial participants.

## Conclusion

In patients with very low LDL-C levels, the lower the level, the more inaccurate the estimate, especially via Friedewald estimation. More often than not, a Friedewald estimate < 40 mg/dL is falsely low, as are the vast majority of values < 25 and < 15 mg/dL. A more accurate method of estimation, such as our novel method, is needed to improve personalized clinical decision-making and assessment of potential adverse effects in the upcoming era of cholesterol management that may witness the attainment of LDL-C levels lower than ever seen before.

## Additional files

Additional file 1: Figure S1. Bland-Altman plot for LDL-C in individuals with very low LDL-C levels. The Bland-Altman plot illustrates the difference against the average of directly-measured (LDLd-C) and estimation methods. Panel A: Friedewald-estimated LDL-C (LDLf-C). Standard Deviation (SD): 6.8 mg/dl. Panel B: Novel method-estimated LDL-C (LDLn-C). Standard Deviation (SD): 4.1 mg/dl. (JPG 114 kb)

Additional file 2: Table S1. Proportions of concordance between Friedewald equation and direct ultracentrifugation LDL-C in individuals with TG < 150, 150–199, and 200–399 mg/dL. (DOCX 144 kb)

Additional file 3: Table S2. Proportions of concordance between novel method and direct ultracentrifugation LDL-C in individuals with TG < 150, 150–199, and 200–399 mg/dL. (DOCX 137 kb)
