# Supplementary figures and images for: Accuracy of low-density lipoprotein cholesterol estimation at very low levels
Source: BMC Med. 2017 Apr 20;15:83. doi: 10.1186/s12916-017-0852-2 (PMC5399386; doi:10.1186/s12916-017-0852-2)

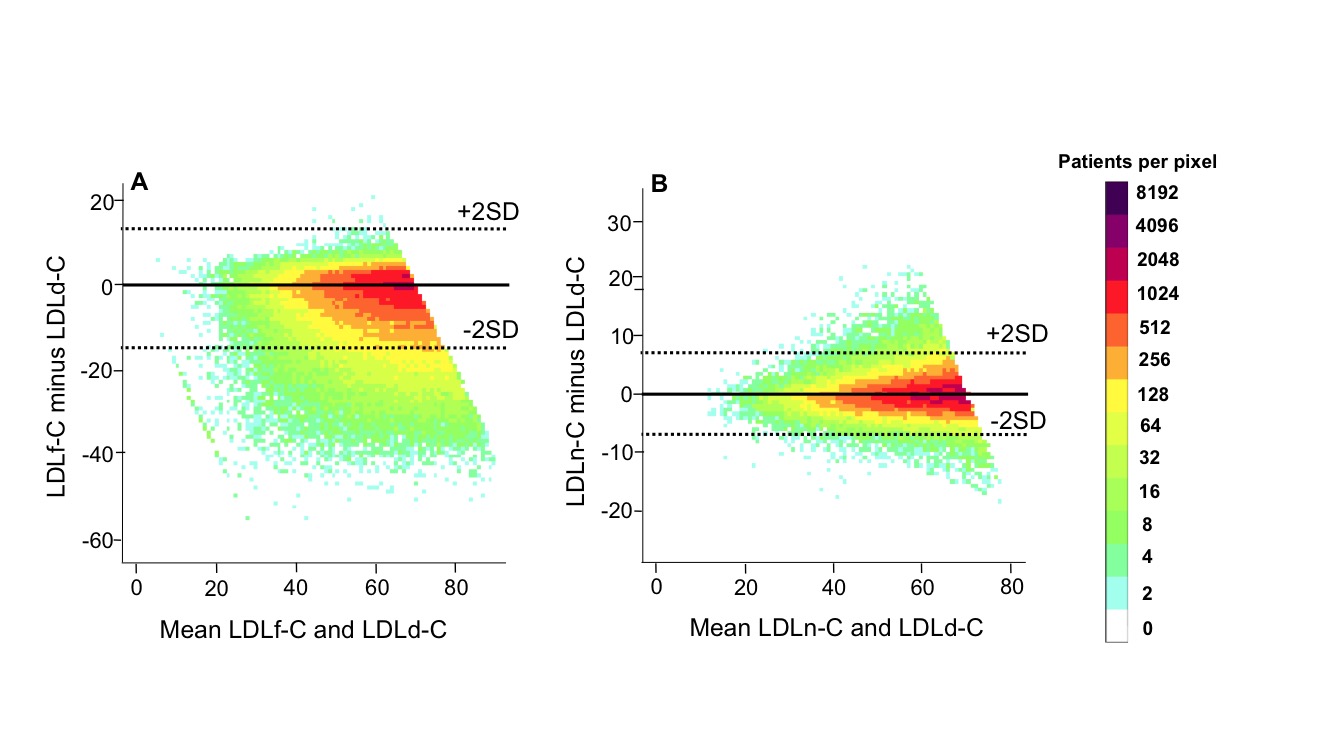

Supplement: Supplementary file 1 — Bland-Altman plot for LDL-C in individuals with very low LDL-C levels. The Bland-Altman plot illustrates the difference against the average of directly-measured (LDLd-C) and estimation methods. Panel A: Friedewald-estimated LDL-C (LDLf-C). Standard Deviation (SD): 6.8 mg/dl. Panel B: Novel method-estimated LDL-C (LDLn-C). Standard Deviation (SD): 4.1 mg/dl. (JPG 114 kb) [file 12916_2017_852_MOESM1_ESM.jpg]
