# Supplementary material for: Accuracy of low-density lipoprotein cholesterol estimation at very low levels
Source: BMC Med. 2017 Apr 20;15:83. doi: 10.1186/s12916-017-0852-2 (PMC5399386; doi:10.1186/s12916-017-0852-2)
Supplement: Supplementary file 2 — Proportions of concordance between Friedewald equation and direct ultracentrifugation LDL-C in individuals with TG < 150, 150–199, and 200–399 mg/dL. (DOCX 144 kb) [file 12916_2017_852_MOESM2_ESM.docx]

**Table S1. Proportions of concordance between Friedewald equation and direct** **ultracentrifugation LDL-C in individuals with TG<150, 150-199 and 200-399 mg/dl**

|  |  | **TG <150** | | | | | | | **TG 150-199** | | | | | | **TG 200-399** | | | | | |
| --- | --- | --- | --- | --- | --- | --- | --- | --- | --- | --- | --- | --- | --- | --- | --- | --- | --- | --- | --- | --- |
|  |  | **Directly-measured LDL-C; mg/dl** | | | | | | | | | | | | | | | | | | |
|  |  | **<15** | **15 to <25** | **25 to <40** | **40 to <50** | **50 to <70** | **≥70** | **<15** | | **15 to <25** | **25 to <40** | **40 to <50** | **50 to <70** | **≥70** | **<15** | **15 to <25** | **25 to <40** | **40 to <50** | **50 to <70** | **≥70** |
| **Friedewald LDL-C; mg/dl** | **<15** | 38 (46.4) | 42 (51.2) | 2  (2.4) | 0  (0.0) | 0  (0.0) | 0  (0.0) | 1 (1.4) | | 33 (45.2) | 39 (53.4) | 0  (0.0) | 0  (0.0) | 0  (0.0) | 3 (0.4) | 41 (6.1) | 323 (48.1) | 201 (29.9) | 103 (15.3) | 1  (0.2) |
|  | **15 to <25** | 26  (3.6) | 343 (47.9) | 347 (48.5) | 0  (0.0) | 0  (0.0) | 0  (0.0) | 0 (0.0) | | 9 (2.8) | 279 (85.8) | 37 (11.4) | 0  (0.0) | 0  (0.0) | 0 (0.0) | 0 (0.0) | 201 (17.8) | 482 (42.8) | 431 (38.3) | 12  (1.1) |
|  | **25 to <40** | 0  (0.0) | 115 (1.5) | 5,168 (68.1) | 2,278 (30.0) | 30  (0.4) | 0  (0.0) | 0 (0.0) | | 0 (0.0) | 363 (15.3) | 1,491 (62.9) | 516 (21.8) | 0  (0.0) | 0 (0.0) | 0 (0.0) | 106 (2.4) | 955 (21.8) | 2,906 (66.3) | 418 (9.5) |
|  | **40 to <50** | 0  (0.0) | 0  (0.0) | 806 (4.3) | 12,421 (65.4) | 5,759 (30.3) | 0  (0.0) | 0 (0.0) | | 0 (0.0) | 19 (0.4) | 529 (11.7) | 3,953 (87.8) | 3  (0.1) | 0 (0.0) | 0 (0.0) | 10 (0.2) | 111 (1.9) | 4,030 (68.0) | 1,767 (29.9) |
|  | **50 to <70** | 0  (0.0) | 0  (0.0) | 5  (<0.1) | 2,043 (2.0) | 87,826 (85.2) | 13,222 (12.8) | 0 (0.0) | | 0 (0.0) | 4 (<0.1) | 63 (0.3) | 9,583 (46.8) | 10,835 (52.9) | 0 (0.0) | 0 (0.0) | 7 (<0.1) | 50 (0.3) | 3,469 (16.5) | 17,478 (83.2) |
|  | **≥70** | 0  (0.0) | 0  (0.0) | 0  (0.0) | 2  (<0.1) | 6,534 (0.8) | 782,042 (99.2) | 0 (0.0) | | 0 (0.0) | 0 (0.0) | 1 (<0.1) | 295 (0.2) | 176,091 (99.8) | 0 (0.0) | 0 (0.0) | 1 (<0.1) | 4 (<0.1) | 291 (0.2) | 153,838 (99.8) |
|  | **Total** | **64** | **500** | **6,328** | **16,744** | **100,149** | **795,264** | **1** | | **42** | **704** | **2,121** | **14,347** | **186,929** | **3** | **41** | **648** | **1,803** | **11,230** | **173,514** |

Numbers shown are n above with row percentages in parenthesis below.

White cells: concordance; Blue cells: discordantly high; Red cells: discordantly low.
