# Supplementary material for: Accuracy of low-density lipoprotein cholesterol estimation at very low levels
Source: BMC Med. 2017 Apr 20;15:83. doi: 10.1186/s12916-017-0852-2 (PMC5399386; doi:10.1186/s12916-017-0852-2)
Supplement: Supplementary file 3 — Proportions of concordance between novel method and direct ultracentrifugation LDL-C in individuals with TG < 150, 150–199, and 200–399 mg/dL. (DOCX 137 kb) [file 12916_2017_852_MOESM3_ESM.docx]

**Table S2. Proportions of concordance between novel method and direct** **ultracentrifugation LDL-C in individuals with TG<150, 150-199 and 200-399 mg/dl**

|  |  | **TG <150** | | | | | | **TG 150-199** | | | | | | **TG 200-399** | | | | | |
| --- | --- | --- | --- | --- | --- | --- | --- | --- | --- | --- | --- | --- | --- | --- | --- | --- | --- | --- | --- |
|  |  | **Directly-measured LDL-C; mg/dl** | | | | | | | | | | | | | | | | | |
|  |  | **<15** | **15 to <25** | **25 to <40** | **40 to <50** | **50 to <70** | **≥70** | **<15** | **15 to <25** | **25 to <40** | **40 to <50** | **50 to <70** | **≥70** | **<15** | **15 to <25** | **25 to <40** | **40 to <50** | **50 to <70** | **≥70** |
| **Novel LDL-C; mg/dl** | **<15** | 57 (63.3) | 33 (36.7) | 0  (0.0) | 0  (0.0) | 0  (0.0) | 0  (0.0) | 1 (12.5) | 7 (87.5) | 0 (0.0) | 0  (0.0) | 0  (0.0) | 0  (0.0) | 3 (15.0) | 13 (65.0) | 4 (20.0) | 0  (0.0) | 0  (0.0) | 0  (0.0) |
|  | **15 to <25** | 7 (1.1) | 405 (63.5) | 226 (35.4) | 0  (0.0) | 0  (0.0) | 0  (0.0) | 0 (0.0) | 25 (35.2) | 46 (64.8) | 0  (0.0) | 0  (0.0) | 0  (0.0) | 0  (0.0) | 15 (19.7) | 58 (76.3) | 3  (4.0) | 0  (0.0) | 0  (0.0) |
|  | **25 to <40** | 0 (0.0) | 62 (0.9) | 5,624 (79.6) | 1,381 (19.5) | 0  (0.0) | 0  (0.0) | 0 (0.0) | 10 (1.3) | 508 (64.5) | 269 (34.1) | 1  (0.1) | 0  (0.0) | 0  (0.0) | 13  (2.1) | 367 (58.2) | 228 (36.2) | 22 (3.5) | 0  (0.0) |
|  | **40 to <50** | 0 (0.0) | 0  (0.0) | 463 (2.6) | 14,042 (79.3) | 3,203 (18.1) | 0  (0.0) | 0 (0.0) | 0 (0.0) | 130 (6.1) | 1,396 (65.0) | 620 (28.9) | 0  (0.0) | 0  (0.0) | 0  (0.0) | 155 (10.3) | 876 (58.2) | 473 (31.4) | 2  (0.1) |
|  | **50 to <70** | 0 (0.0) | 0  (0.0) | 15 (<0.1) | 1,317 (1.3) | 91,336 (92.4) | 6,200 (6.3) | 0 (0.0) | 0 (0.0) | 20 (0.1) | 443 (3.4) | 11,435 (86.9) | 1,259 (9.6) | 0  (0.0) | 0  (0.0) | 57 (0.5) | 648 (5.8) | 8,214 (73.7) | 2,225 (20.0) |
|  | **≥70** | 0 (0.0) | 0  (0.0) | 0  (0.0) | 4 (<0.1) | 5,610 (0.7) | 789,064 (99.3) | 0 (0.0) | 0 (0.0) | 0 (0.0) | 13 (<0.1) | 2,291 (1.2) | 185,670 (98.8) | 0  (0.0) | 0  (0.0) | 7 (<0.1) | 48 (<0.1) | 2,521 (1.5) | 171,287 (98.5) |
|  | **Total** | 64 | 500 | 6,328 | 16,744 | 100,149 | 795,264 | 1 | 42 | 704 | 2,121 | 14,347 | 186,929 | 3 | 41 | 648 | 1,803 | 11,230 | 173,514 |

Numbers shown are n above with row percentages in parenthesis below.

White cells: concordance; Blue cells: discordantly high; Red cells: discordantly low.
